# Multi-Component Xinjiang Basil Essential Oil Mitigates Alzheimer’s Disease-like Pathology Through Amyloid and Neuroinflammatory Modulation

**DOI:** 10.3390/nu18183058

**Published:** 2026-09-18

**Authors:** Sendaer Hailati, Yibulayin Yasheng, Yiqing Yin, Chuhan Jiang, Yuhang Liu, Wenxi Ding, Dilihuma Dilimulati, Alhar Baishan, Yipaerguli Paerhati, Alifeiye Aikebaier, Arzuye Dolkun, Wenting Zhou

**Affiliations:** 1 Department of Pharmacology, School of Pharmacy, Xinjiang Medical University, Urumqi 830017, China; 2 Xinjiang Key Laboratory of Natural Medicines Active Components and Drug Release Technology, Urumqi 830017, China; 3 Xinjiang Key Laboratory of Biopharmaceuticals and Medical Devices, Urumqi 830017, China; 4 Engineering Research Center of Xinjiang and Central Asian Medicine Resources, Ministry of Education, Urumqi 830017, China

**Keywords:** basil essential oil, Alzheimer’s disease, neuroinflammation, IκB-α/NF-κB signaling

## Abstract

**Background**: Basil (*Ocimum basilicum* L.) is a herbaceous plant belonging to the family Lamiaceae. It is not only edible, but it also has nutritional value and can be used for medicinal purposes. Previous studies suggest that basil-derived extracts may protect neurons from injury. **Objectives**: Our research aimed to investigate whether basil essential oil (BEO) from Xinjiang has a neuroprotective effect in Alzheimer’s disease (AD)-like pathological mouse model and to elucidate the mechanism underlying this effect. **Methods**: The essential oil was extracted using steam distillation. UHPLC–MS/MS technology was used to identify constituents detected in brain tissue following intranasal administration. In the experiment, BEO was administered intranasally to APP/PS1 mice for 4 weeks, and its therapeutic effects on AD-like pathological features were evaluated through behavioral and biochemical assessments. **Results**: The results showed that there were 787 compounds in the BEO, of which 30 putatively annotated compounds were detected in brain tissue after intranasal administration. Most of these were prenol lipids (36%). BEO markedly enhanced spatial learning and cognitive abilities in APP/PS1 mice, while also reducing neurodegeneration within the hippocampus and decreasing Aβ_1–40_ and Aβ_1–42_ levels. BEO additionally lowered the levels of IL-1β, IL-6, and TNF-α and inhibited the activation of astrocytes and microglia. BEO exerted its neuroprotective effects by reducing the activation of the IκB-α/NF-κB pathway. **Conclusions**: BEO exerted neuroprotective-like effects in this AD mouse model accompanied by anti-inflammatory activity and suppression of amyloid-β (Aβ) accumulation, and BEO treatment was associated with reduced IκB-α/NF-κB pathway activation. This research demonstrated that BEO might serve as a natural treatment against AD.

## 1. Introduction

Alzheimer’s disease is a progressive neurodegenerative disorder. It is the type of dementia that most commonly affects the elderly. Its pathology involves Aβ aggregation, tau hyperphosphorylation and tau-associated neurotoxicity, chronic neuroinflammation, oxidative stress, mitochondrial impairment, and dysregulation of neuronal signaling, and gradual synaptic failure [1]. These problems do not occur independently. They are related to aging, poor metabolic function, vascular dysfunction, genetic predisposition to the disease, and living environment factors. Therefore, it is extremely difficult to control AD by modulating a single therapeutic target [2].

Recent approaches to treating diseases have mainly focused on how to eliminate Aβ. These methods have shown that amyloid proteins can be modified. However, their clinical benefit remains limited in many patients, and safety concerns also remain [3,4]. This has led researchers to consider whether there could be a way to address multiple pathological processes simultaneously. Among these events, neuroinflammation has emerged as an important area of investigation. Initially, these activated microglia and astrocytes are quite effective at clearing Aβ. However, problems arise if these cells remain in an activated state. Excessive cytokine release can lead to synaptic dysfunction, which in turn causes damage to neurons [5,6,7]. The response of glial cells is not static but depends on the specific circumstances. Whether at different stages of a disease or influenced by signals from the surrounding environment, these reactive astrocytes and microglia can sometimes contribute positively, but can also exert detrimental effects [8,9,10]. The NF-κB signaling pathway is a major regulator of inflammatory signaling in regulating inflammation. Once activated, it causes the body to produce various inflammatory factors such as IL-1β, IL-6, and TNF-α. Additionally, the NF-κB signaling pathway is also closely related to the toxicity caused by Aβ and tau proteins [11,12]. If we could target this pathway, it might be possible to break the vicious cycle between amyloid deposition, activated glial cells, and damaged neurons. Notably, six-month-old APP/PS1 mice represent a well-established pathological stage of AD. At this age, the mice spontaneously develop significant cerebral amyloid deposition and obvious cognitive deficits, which have been consistently validated in previous studies [13,14]. Pathological onset slightly varies by transgenic strain and experimental conditions, but robust AD-like lesions are universally present in 6-month-old APP/PS1 mice.

Basil (*Ocimum basilicum* L.) is a member of the *Lamiaceae* family, a well-known edible spice and medicinal herb. It is widely distributed in Iran and India, as well as in the Xinjiang, Yunnan, and Guangxi regions of China. With its rich content of bioactive nutrients, this plant offers multifaceted benefits in disease prevention and physiological regulation, holding tremendous potential for nutritional and pharmaceutical development. Our previous research has confirmed that Xinjiang basil, as a traditional Uyghur medicinal plant, has preventive and protective effects against cardiovascular diseases. It regulates the expression of epoxydase isoenzymes and thrombo-related prostaglandins, inhibits thrombus formation, and ultimately reduces the risk of cardiovascular events [15]. Recent research indicates that the bioactive components of the *Ocimum* genus act on the nervous system through nutritional intervention, restoring neural function and alleviating neuronal damage, thus demonstrating significant neuroprotective potential [16]. Another pharmacological study confirmed that trimethoxyflavone, isolated from basil leaves, can modulate various physiological pathways, improve long-term memory, and were associated with anti-acetylcholinesterase (AChE), antioxidant, anti-inflammatory, and anti-apoptotic activities, thereby helping to prevent neurological diseases [17]. Nevertheless, the preventive and therapeutic effects of basil essential oil (BEO)on AD remain unclear. Specifically, there is currently insufficient evidence to elucidate the identifiable components of BEO in the rat brain, the efficacy of BEO in improving behavioral performance in AD-like pathological mouse model, or its regulatory effects on AD-related pathological alterations and inflammatory responses in vivo. We selected intranasal delivery for this study. Volatile lipophilic BEO constituents may gain access to brain tissue after intranasal administration [18]. It has been reported that the olfactory–trigeminal pathway can bypass the hepatic first-pass metabolism and achieves target brain exposure at much lower doses compared with oral application [19]. However, the exact route of brain exposure was not established in the present study.

In this study, BEO was prepared by distillation and then administered intranasally to APP/PS1 mice. First, we analyzed the chemical composition of the extract and employed UHPLC-MS/MS to identify the compounds detectable in brain tissue. Then we evaluated its effects on spatial learning and spatial reference memory, hippocampal morphology, Aβ deposition, glial activation, inflammatory cytokines, and IκB-α/NF-κB signaling. This design aimed to characterize whether BEO could alleviate AD-like pathology in APP/PS1 mice and to explore observational changes within the NF-κB/IκB-α signaling axis.

## 2. Materials and Methods

### 2.1. Reagents and Chemicals

Dichloromethane, acetonitrile, methanol, formic acid, ethanol, xylene, and PVDF membranes (0.45 μm) were purchased from Millipore (Boston, MA, USA); Tween 80, 4% paraformaldehyde tissue fixative, Cocktail protease inhibitor, BCA protein quantification kit, 4× loading buffer, and SDS gel preparation kit were acquired from Solarbio (Beijing, China); RIPA tissue lysis buffer and trichrome protein pre-stained marker were acquired from Thermo Fisher (Waltham, MA, USA); hematoxylin stain, eosin stain, toluidine blue stain, and 1× TBST buffer were acquired from Servicebio (Wuhan, China); mouse IL-1β, IL-6, TNF-α, Aβ_1–40_, and Aβ_1–42_ detection kits were purchased from Elabscience (Wuhan, China); a series of rabbit primary antibodies: anti-IκB-α (Cat No. 82349-1-RR), anti-p-IκB-α (Ser32/36, Cat No. 82349-1-RR), anti-NF-κB p65 (Cat No. 80979-1-RR), anti-p-NF-κB p65 (Ser468, Cat No. 82335-1-RR), anti-β-actin (Cat No. 81115-1-RR), anti-Aβ_1–42_ (Cat No. 25524-1-AP), anti-GFAP (Cat No. 16825-1-PBS), anti-Iba-1 (Cat No. 10904-1-PBS), and HRP-conjugated goat anti-rabbit IgG secondary antibody (Cat No. SA00001-2) were purchased from Proteintech (Wuhan, China); skim milk powder was sourced from Biofroxx (Einhausen, Germany); donepezil hydrochloride was purchased from Chongqing Zhi’en Pharmaceutical Co., Ltd. (Chongqing, China).

### 2.2. Preparation and Chemical Characterization of BEO

The Xinjiang basil was formally identified and authenticated by Prof. Palida Abulizi (Department of Natural Medicinal Chemistry and Pharmacognosy, School of Pharmacy, Xinjiang Medical University). A voucher specimen (NO.TCMEHSM2013_ 102) has been stored in the Ethnical Herbs Specimen Museum of Traditional Chinese Medicines at Xinjiang Medical University. And the fresh aerial parts of Xinjiang basil were harvested by members of our research group in Turpan, Xinjiang, China, on 23 August 2024. The 74.6 kg fresh aerial parts of basil were transferred into a round-bottom flask, mixed with distilled water at a solid-to-liquid ratio of 1:10 (g/mL), and several boiling chips were added to prevent liquid bumping. Subsequently, hydro-distillation was performed using a standard volatile oil determination apparatus with circulating cooling water in a lower-in and upper-out mode. The distillation process was timed from the moment the first drop of distillate dripped from the condenser outlet, and continuous distillation was maintained for 3 h. After distillation completion, the system was cooled naturally to room temperature. The distillate was collected and extracted three times with an equal volume of diethyl ether. After combining the diethyl ether extracts and recovering the diethyl ether, a deep yellow, oily substance with a rich aroma was obtained. A total of 54.5 g (60 mL) of BEO was obtained, corresponding to an essential oil yield of approximately 0.073% (*w*/*w*). The resulting BEO was transferred into amber glass vials, sealed, and stored at 4 °C for subsequent experiments.

BEO was solubilized in 1% Tween-80 and was further diluted with 0.9% saline to the desired concentrations. For chemical characterization, 1 mL of the BEO solution was mixed with 3 mL of ethanol; the mixture was fully vortex-mixed, and was sonicated at ambient temperature for 10 min. The mixture was then stored at 4 °C for 12 h. The mixture was centrifuged at 4000× *g* for 10 min at 4 °C, and the supernatant was harvested. Ethanol was evaporated under a nitrogen stream. Before UHPLC-MS/MS measurement, the sample volume was reconstituted using pre-chilled methanol–water (4:1, *v*/*v*), and was filtered using a 0.22 μm membrane filter. Thereafter, the sample was injected (10 μL) for LC-MS/MS analysis, and three technical replicates were run for each sample.

### 2.3. Animals and Treatments

Two-month-old male SPF (Specific Pathogen-Free)-grade SD rats were purchased from the Animal Experimentation Center of Xinjiang Medical University. Six-month-old male SPF-grade APP/PS1 double-transgenic AD model mice and age-matched male SPF-grade C57BL/6J mice were purchased from Jiangsu Huachuang Xinnuo Pharmaceutical Technology Co., Ltd. (Taizhou, China). Certificate of Animal Quality No.: B202408060010. Animals were housed under standard laboratory conditions. All animals were acclimated to the experimental conditions for 7 days prior to the experiment, with free access to food and water.

To evaluate the constituents detected in brain tissue following intranasal administration, male SD rats were randomly divided into 2 groups (*n* = 3 per group); the vehicle control group received an equivalent dose of sterile saline intranasally, and the BEO treatment group received 0.12 mL/kg of BEO intranasally. The total volume was divided into small aliquots and alternately instilled into each nostril to ensure optimal absorption and minimize loss.

The APP/PS1 mice were divided randomly into 5 groups (*n* = 9 per group): the AD model group (Model) was administered 0.02 mL of sterile saline; the positive control group (Donepezil) was administered 0.65 mg/kg donepezil intranasally; the BEO low-dose-treated group (BEO-L) was administered 1 mL/kg 1%BEO; the BEO medium-dose-treated group (BEO-M) was administered 1 mL/kg 2%BEO; the BEO high-dose-treated group (BEO-H) was administered 1 mL/kg 3%BEO; and 9 male C57BL/6J mice in the blank control group (Blank) were administered 0.02 mL of sterile saline. BEO and sterile saline were administered via intranasal delivery once daily for 4 weeks. BEO was slowly instilled into the bilateral nostrils using a micropipette in alternating drops to ensure proper absorption. The primary outcome measure of this study was spatial cognitive performance evaluated by the Morris water maze (MWM) test (escape latency, platform-crossing number, and target-quadrant residence time). Biochemical and histopathological markers including Aβ burden, glial activation and neuroinflammatory cytokines were defined as secondary outcome measures. After 7-day acclimatization, APP/PS1 mice were randomly allocated into experimental groups using the random number-generation method. To minimize confounding factors, cage positions were rotated twice weekly during drug administration. The testing order of animals in behavioural assays was also systematically rotated across groups to reduce time-of-day and handling-order bias.

All animal experimentation protocols were approved by the Laboratory Animal Ethics Committee of Xinjiang Medical University (Ethical Approval No.: IACUC-20230309-154).

### 2.4. Collection and Preparation of Rat Brain Tissue Samples

Given that AD-related pathological targets reside within the brain, we aimed to characterize the constituents detectable in brain tissue following intranasal administration of BEO. One hour following the final dose, rats were anesthetized by intraperitoneal injection of 1% sodium pentobarbital (0.04 mL/kg) and were sacrificed. Whole-brain tissues were carefully dissected, rinsed with ice-cold saline, blotted dry, weighed, and stored at −80 °C. Brain tissue samples, two stainless-steel grinding beads, and 400 μL pre-chilled methanol–water mixture (4:1, *v*/*v*) were loaded into grinding tubes. Samples were homogenized under low-temperature conditions for 5 min. An additional 600 μL of pre-chilled methanol–water (4:1, *v*/*v*) was added; the suspension was thoroughly mixed and was subjected to ice-bath sonication for 20 min, followed by incubation at −20 °C for 1 h. Samples were centrifuged at 16,000× *g* for 20 min at 4 °C. The supernatant was collected and was concentrated to complete dryness in a vacuum concentrator. Prior to UHPLC-MS/MS, samples were reconstituted, were centrifuged at 20,000× *g* for 15 min at 4 °C, and were aliquoted for instrumental measurement.

### 2.5. UHPLC-MS/MS Analysis

The sample was placed in an automatic sampler where the temperature remained at 4 °C. It was then analyzed using a SHIMADZU-LC30 UHPLC system. The column temperature was set at 40 °C, and the flow rate was set to 0.3 mL/min. Mobile phase A consisted of an aqueous solution containing 0.1% formic acid, while mobile phase B was an acetonitrile solution containing 0.9% formic acid. Gradient elution was then performed. Mass spectrometry analysis was performed using a TripleTOF 6600 system (AB Sciex, Framingham, MA, USA), applying positive and negative ion modes with electrospray ionization. Source gas parameters are shown in Table 1. Data was acquired over a 30 min period, with the time-of-flight mass spectrometry (TOF MS) scanning range set at 90–1500 *m*/*z* and the product ion scanning range at 50–1500 *m*/*z*. The 18 ions with the highest intensity (>100 cps) were selected from the TOF MS scan and fragmented using information-dependent acquisition (IDA), with dynamic background subtraction enabled.

Using MS-DIAL 5.5 software, we processed the raw mass spectrometry data. Key steps in this process included alignment of peaks, correction of retention times, and extraction of peak areas. The identification criteria for features putatively annotated in brain tissue following intranasal administration of BEO were: Primary MS tolerance < 0.01 Da, secondary MS tolerance < 0.02 Da, and a secondary MS score > 70%. All annotations rely solely on spectral-library matching; no authentic reference standards were applied for compound confirmation. No individual biological-activity assessment was performed for these annotated features in this study. Putatively annotated features detected in brain tissue were classified and used R 4.3.1 software to visualize the distribution of these compounds at the superclass and class levels.

### 2.6. Behavioral Assessments

#### 2.6.1. Morris Water Maze (MWM) Test

In order to examine the behavioral effects of BEO on spatial learning and spatial reference memory in APP/PS1 mice, six experimental groups underwent the MWM test, which consisted of a five-day platform-hidden training phase and a one-day probe test. The experimental apparatus consisted of a circle-shaped pool (110 cm × 48 cm) filled with water, a circle-shaped hidden platform (10 cm × 28 cm), and the WMT-100S video-tracking analysis system (Chengdu Techman Software Co., Ltd., Chengdu, China) for automatic trajectory recording. Before the experiment began, the pool was filled with water maintained at 25 °C, and titanium dioxide was added to produce an opaque milky white water surface. The water level had to be 1 to 1.5 cm higher than the surface of the platform. The pool was virtually divided into four quadrants with the platform right in the center of the target quadrant (Quadrant III). During the hidden-platform acquisition phase, each mouse received one trial per day for five consecutive days, with no repeated trials within a single day. All animals were gently released facing the pool wall from the quadrant diagonally opposite to the target platform; randomized starting positions were not adopted in this study. This fixed-starting-position paradigm was used to assess spatial memory for platform location, and progressive reduction in escape latency across training days was taken as evidence of spatial learning. The trajectories were automatically recorded by the system. Mice were allowed to swim for up to 60 s to find the hidden platform. If the mouse successfully reached the platform and stayed on it for at least 5 s, the entire process from entering the pool to reaching the platform was recorded as the escape latency. However, the escape latency was recorded as 60 s in cases of failure to reach the platform within 60 s. At this point, the mouse would be guided back onto the platform and permitted to stay on it for 10 s. During the five-day experimental period, the escape latency and swimming trajectories were automatically recorded daily. Finally, a WMT-100S video-tracking analysis system (Chengdu Techman Software Co., Ltd., Chengdu, China) was used to process and visualize the data.

Following hidden platform training, the platform in Quadrant III was removed to conduct the probe test. Each mouse completed only one single probe trial. The mice entered the platform sequentially from a randomly selected starting quadrant among Quadrants I, II, and IV, facing the wall of the pool. During the 60 s test period, recordings were made of the time mice spent in the target quadrant as well as the number of crossings over the original platform. Such data served to evaluate the spatial learning and spatial reference memory abilities of the mice. All data were exported to Excel for further statistical analysis.

#### 2.6.2. Open Field Test

The Open Field Test is designed to assess spontaneous exploratory behavior in mice. Individual mice were placed in a square test chamber (50 cm × 50 cm × 50 cm) and allowed to move freely for 3 min. The entire process was recorded using an automated video system, and the total distance traveled, time spent in the central area, and mean speed were quantified. Between each trial, the testing arena was completely cleaned with 75% alcohol to eliminate residual odors and prevent olfactory cues left by previous mice from influencing subsequent mice. All data were exported to Excel for further statistical analysis.

Single-blind assessment was applied during behavioural data acquisition and analysis. The experimenter performing behavioural scoring and trajectory analysis was kept unaware of group assignments throughout the MWM and open-field tests. Group identity was known only to the researcher responsible for animal allocation and drug administration.

### 2.7. Histopathological Examination

After the behavioral assessment was completed, the mice received sodium pentobarbital anesthesia and were sacrificed. Following careful dissection, brain and liver tissues were fixed in 4% paraformaldehyde at ambient temperature for 24 h. Fixed tissues were processed through a series of steps, paraffin-embedded, and finally sectioned into 5 μm-thick sections for histological examination.

#### 2.7.1. Hematoxylin–Eosin (H&E) Staining

Paraffin-embedded sections should first be deparaffinized with xylene and were rehydrated through graded ethanol series. The sections were stained with hematoxylin and then were counterstained with 0.5–1% Eosin. After staining, the sections needed to be dehydrated and made transparent and finally covered with a sealing material. The images were captured using an optical microscope. In addition, a qualitative analysis of the organizational morphology was conducted to understand the arrangement of neurons and the characteristics of liver structure.

#### 2.7.2. Nissl Staining

After deparaffinization, the brain sections were stained with toluidine blue to highlight neuronal cell bodies and Nissl bodies, focusing on the observation of the hippocampal CA1 and CA3 regions. Images were captured under an optical microscope.

#### 2.7.3. Immunohistochemistry (IHC)

Brain sections were deparaffinized in a concentration gradient of organic solvents and subsequently underwent antigen retrieval in a microwave oven. The sections were exposed to 3% hydrogen peroxide for 25 min at ambient temperature in the dark to inactivate endogenous peroxidase. Sections were incubated with primary antibodies at 4 °C overnight: anti-Aβ_1–42_ (1:100), anti-GFAP (1:200, astrocyte marker), and anti-Iba-1 (1:200, microglial marker). Following washing, secondary antibodies labeled with HRP were added, with DAB used for developing. Nuclear counterstaining was performed with hematoxylin. After a stepwise acid–alcohol treatment, dehydration, and clearing, the sections were covered with a coverslip. For quantitative analysis of Aβ_1–42_, GFAP, and Iba-1 immunoreactivity, coronal brain sections spanning bregma −1.70 mm to −2.30 mm were selected according to the Paxinos and Franklin mouse brain atlas. Three to five spatially spaced sections containing hippocampal regions were analyzed per mouse. Three random microscopic fields were captured for each target hippocampal region under identical fixed magnification, and all camera and illumination settings were kept constant across experimental groups. Image analysis was performed using ImageJ 1.54P software with a fixed global threshold, and this identical threshold setting was applied uniformly to all groups to minimize subjective bias. IHC image semi-quantitative analysis was performed by an investigator blinded to experimental group information.

### 2.8. Biochemical Assays

Frozen hippocampal tissues were resuspended in ice-cold PBS and were homogenized. Homogenates were centrifuged at 13,000× *g* for 10 min, and supernatants were harvested. IL-1β, IL-6, TNF-α, Aβ_1–40_, and Aβ_1–42_ concentrations were quantified using commercial ELISA kits following the manufacturer’s instructions.

### 2.9. Western Blot Analysis

The frozen hippocampal tissues were thawed, weighed, and were homogenized in RIPA lysis buffer supplemented with a 0.1% protease inhibitor mixture. Lysates were centrifuged at 12,000× *g* for 15 min at 4 °C. Supernatants were collected, and total protein concentration was determined using the BCA assay. Equal amounts of protein (40 µg per lane) were loaded for SDS-PAGE separation. Following separation, the proteins were transferred to a PVDF membrane and membranes were incubated in 5% non-fat milk at 4 °C for 2 h.

The membrane was incubated overnight at 4 °C with antibodies against NF-κB, p-NF-κB, IκB-α, p-IκB-α, and β-actin diluted at a 1:1000 ratio. Following washing, the membrane was exposed to HRP-labeled goat anti-rabbit secondary antibody diluted 1:1000 for 2 h at ambient temperature. Adding chemiluminescent substrate at the recommended concentration developed the membrane, and images of the protein bands were captured using the GeneSys chemiluminescence imaging system. Band intensities were quantified using ImageJ software, standardizing expression levels of the target proteins against β-actin as an internal control. Western blot band quantification was conducted under single-blind conditions without knowledge of mouse group assignment. All experiments included at least three biologically independent animals per group. Each animal underwent separate tissue processing and western blot measurement, and results were presented relative to baseline control samples.

### 2.10. Statistical Analysis

The data were presented as mean ± standard deviation (SD). Statistical analysis was accomplished through one-way analysis of variance (ANOVA), two-way repeated-measures ANOVA, followed by Tukey’s post hoc test and Tukey’s multiple comparison test. Differences were considered statistically significant at *p* < 0.05.

## 3. Results

### 3.1. Chemical Profiling and Putatively Annotated Compounds Were Detected in Brain Tissue

**Figure 1:**
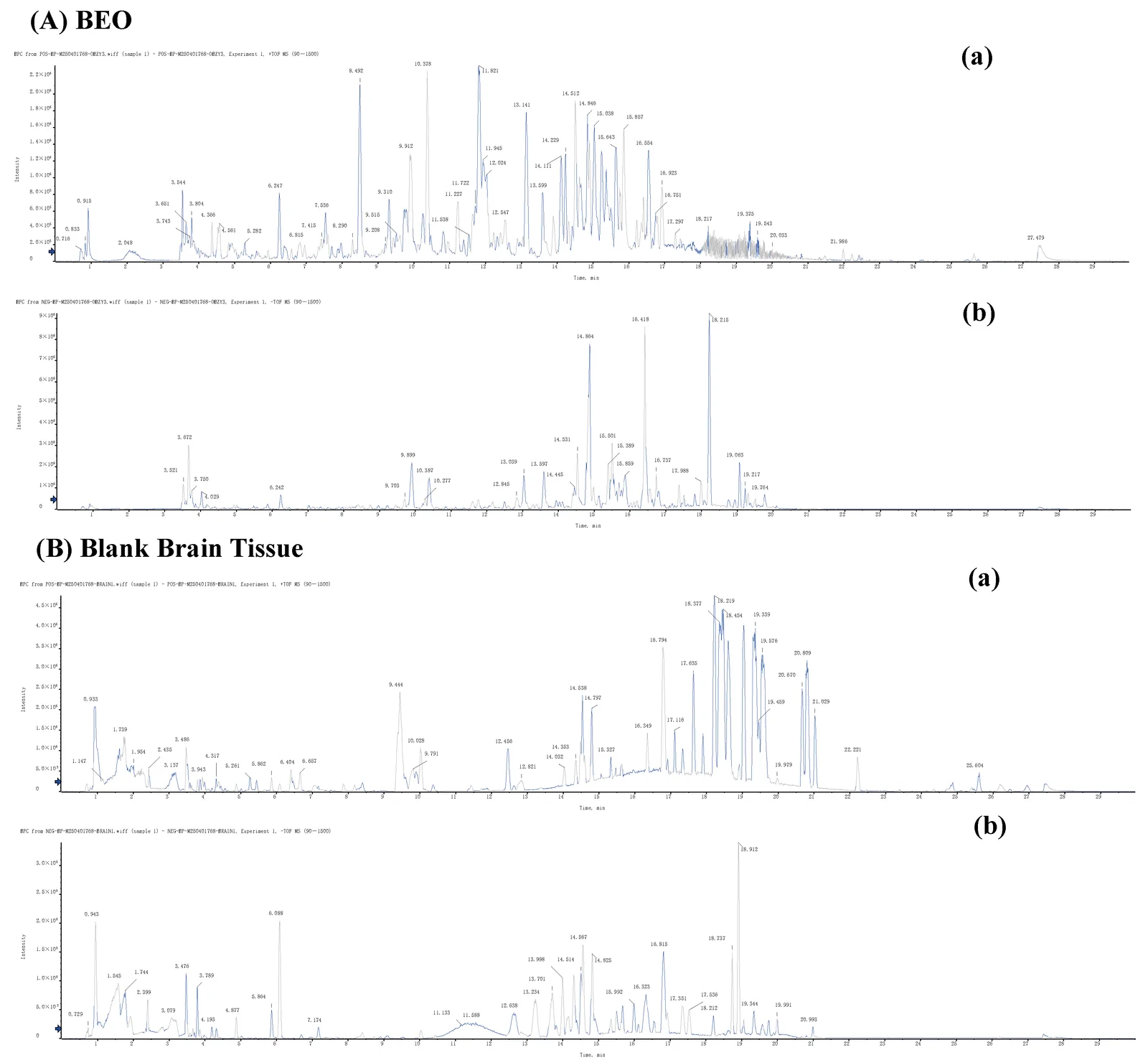

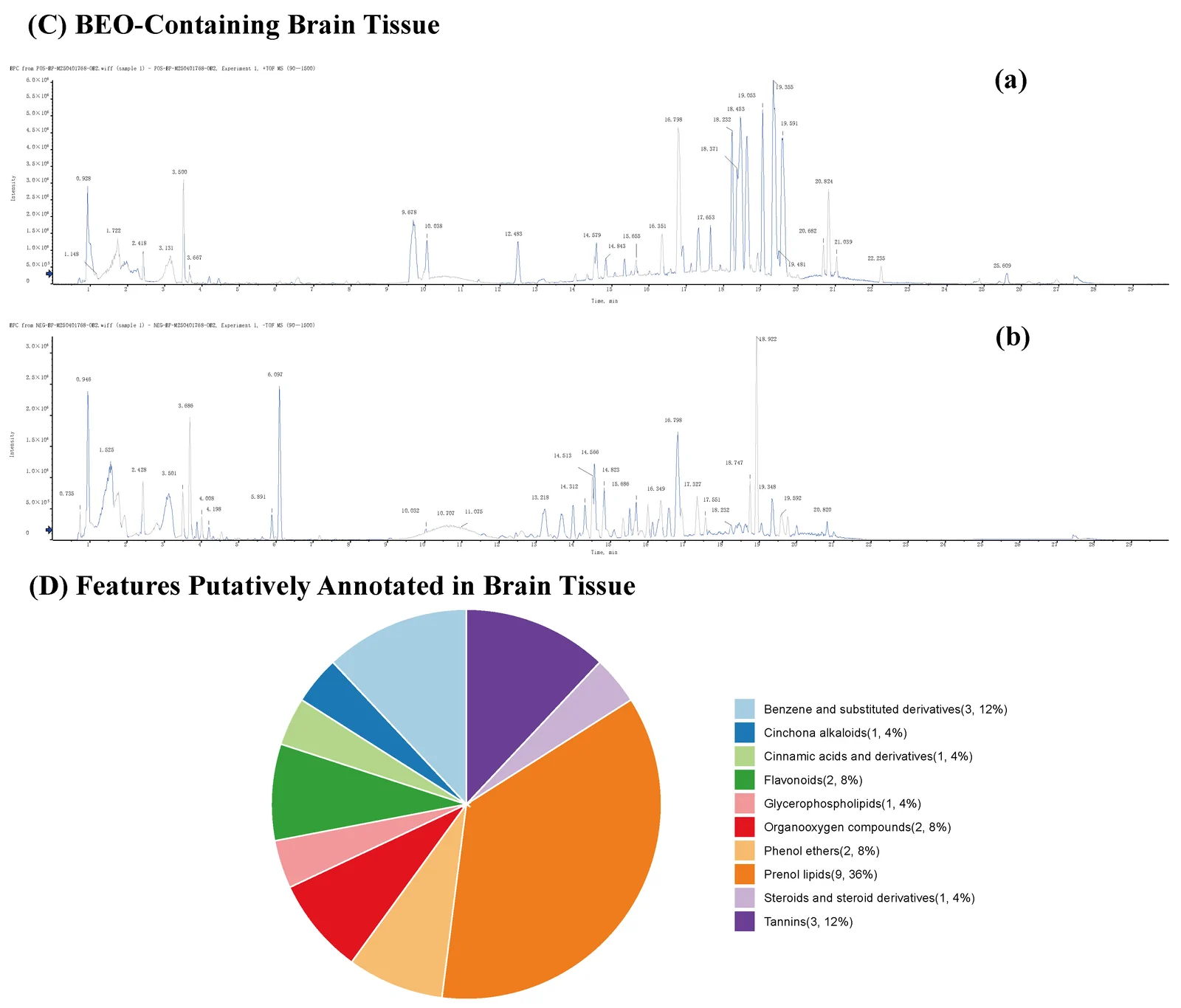
UHPLC-MS/MS-based chemical profiling of BEO and features detected in brain tissue after intranasal BEO administration. Representative total ion chromatograms acquired in positive-ion (**a**) and negative-ion (**b**) modes are shown for (**A**) BEO, (**B**) blank brain tissue, and (**C**) brain tissue collected after BEO administration. The blue arrow indicates the increasing direction of retention time on the x-axis. (**D**) Chemical classification of features putatively annotated in brain tissue following BEO intranasal administration. These spectral features were detected in brain tissue following intranasal administration of BEO. All chemical classifications are based on putative spectral-library annotations without authentic-standard confirmation.

To investigate the constituents that can be detected after intranasal administration of BEO, comparisons were conducted between the herbal control and brain tissues from the BEO-treated group and the untreated group. Total ion chromatograms for each sample were acquired in both positive and negative ion modes (Figure 1A–C). Identification was based on the compounds’ retention times, molecular weights, and ionization patterns. UHPLC–MS/MS analysis revealed a total of 898 compounds, of which 787 were identified as constituents of BEO. Notably, 30 compounds were detected in the brain following intranasal administration of BEO (Table 2). The putatively annotated compounds were primarily classified as prenol lipids (36%), followed by benzene/substituted derivatives, and tannins (12% each). Flavonoids, phenolic ethers, and organic oxygen-containing compounds each accounted for 8% of the total, while trace compounds including cinchona alkaloids, cinnamic acid and its derivatives, glycerophospholipids, and steroids/steroid derivatives each accounted for 4% (Figure 1D). These putatively annotated spectral features were observed in brain tissue. However, individual compound bioactivity and the exact route for brain exposure remain unproven in the current study.

### 3.2. BEO Improved the Cognitive Performance in APP/PS1 Mice

**Figure 2:**
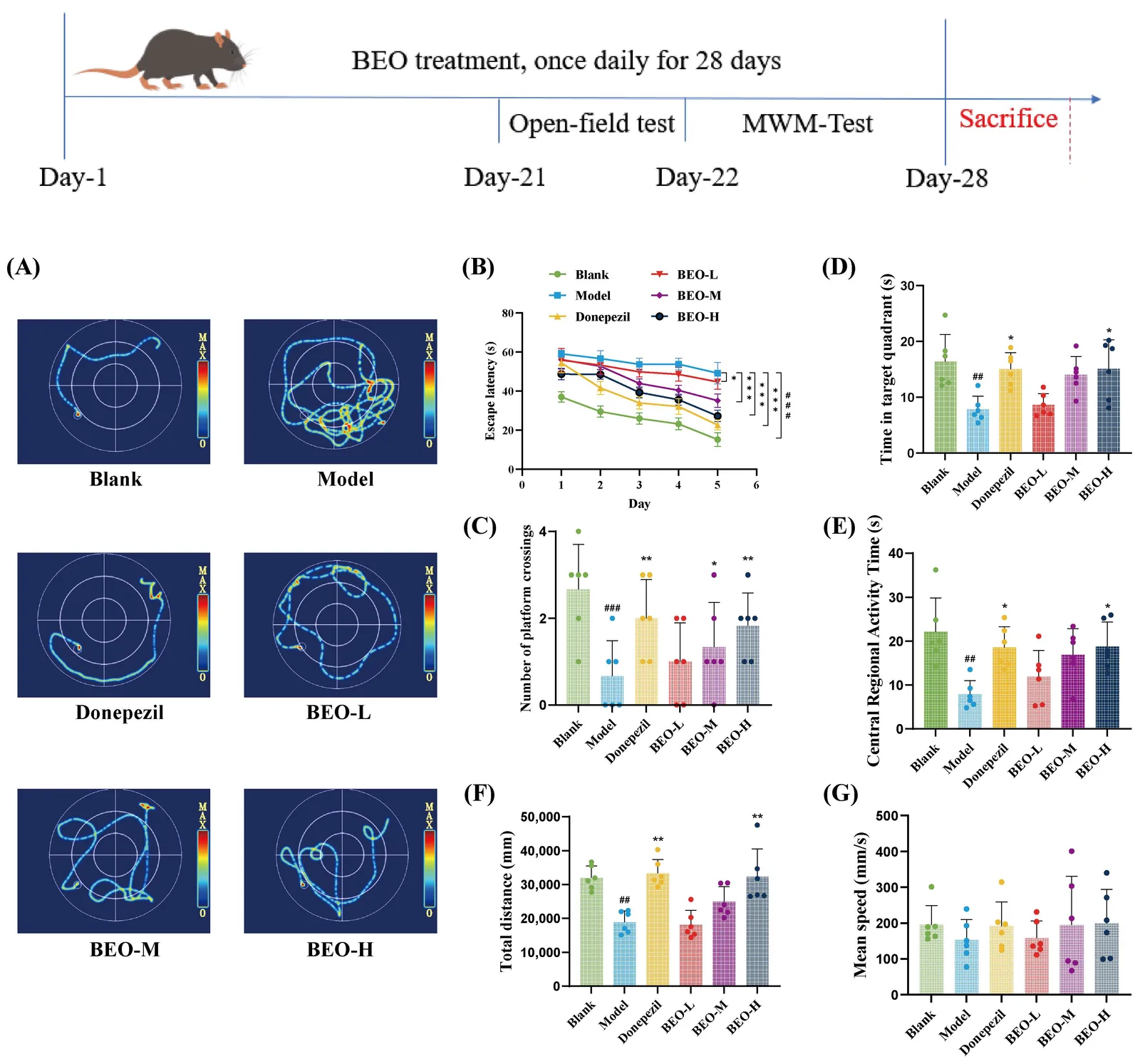
BEO Intranasal Treatment Timeline and Behavioral Outcomes. Intranasal BEO treatment improved spatial learning and spatial reference memory performance in the Morris water maze (MWM) and the Open Field test. (**A**–**D**) Morris water-maze behavioural outcomes: (**A**) Representative swimming-trajectory heat-maps during the probe trial; (**B**) Escape latency recorded across the five-day hidden-platform acquisition phase; (**C**) Number of platform crossings during the probe trial; (**D**) Time spent within the target quadrant in the probe trial. (**E**–**G**) Open-field-test behavioural outcomes: (**E**) Time spent in the central region; (**F**) Total travel distance; (**G**) Mean swimming speed. Data are presented as mean ± SD, *n* = 6 biological replicate mice per group. For escape latency across the 5-day hidden-platform acquisition phase (**B**), two-way repeated-measures ANOVA was performed with treatment, day, and treatment × day interaction as fixed factors, followed by post-hoc comparisons with appropriate correction for repeated-measures data. Single-time-point endpoints from the probe trial (platform crossings, target-quadrant time, (**C**,**D**)) and open-field indices (**E**–**G**) were analyzed by one-way ANOVA followed by Tukey’s multiple-comparison test. ^##^ *p* < 0.01 and ^###^ *p* < 0.001 vs. Blank; * *p* < 0.05, ** *p* < 0.01, and *** *p* < 0.001 vs. Model.

Following four weeks of intranasal BEO treatment, the effects on behavioral performance in spatial learning and exploration were evaluated through the Morris water maze (MWM) and open-field tests. During the MWM learning phase, model group mice displayed more disorganized swimming trajectories than the Blank group (Figure 2A), and showed significantly increased escape latency (Figure 2B) (*p* < 0.001). The number of platform crossings (Figure 2C) and the time spent in the target quadrant were both significantly reduced (Figure 2D) (*p* < 0.01–0.001). However, both mice treated with donepezil and those treated with medium- and high-dose BEO displayed increasingly linear swimming trajectories, a significantly shorter escape latency (*p* < 0.001), and a gradual increase in the number of platform crossings (*p* < 0.01). Even with low-dose BEO, we observed a significantly reduced escape latency (*p* < 0.05). In addition, both the Donepezil group and the high-dose BEO group showed a significantly prolonged time spent in the target quadrant (*p* < 0.05).

In the Open Field test, the mice in the Model group spent less time in the central region (Figure 2E) and covered a shorter total distance (Figure 2F) (*p* < 0.01). The software excludes stationary periods when computing average speed. Total distance is calculated across the full 3 min test window, while maintaining unchanged average speed (Figure 2G). Both donepezil and intranasal BEO treatment at medium- and high-doses significantly increased central region activity and total distance moved (*p* < 0.05–0.01), reflecting enhanced exploratory behavior. Collectively, the findings indicated that BEO improves spatial learning, spatial reference memory, and spontaneous activity in APP/PS1 mice, with the most remarkable effects observed at medium- and high-doses of BEO.

### 3.3. BEO Attenuates Hippocampal Neuronal Damage in APP/PS1 Mice

**Figure 3:**
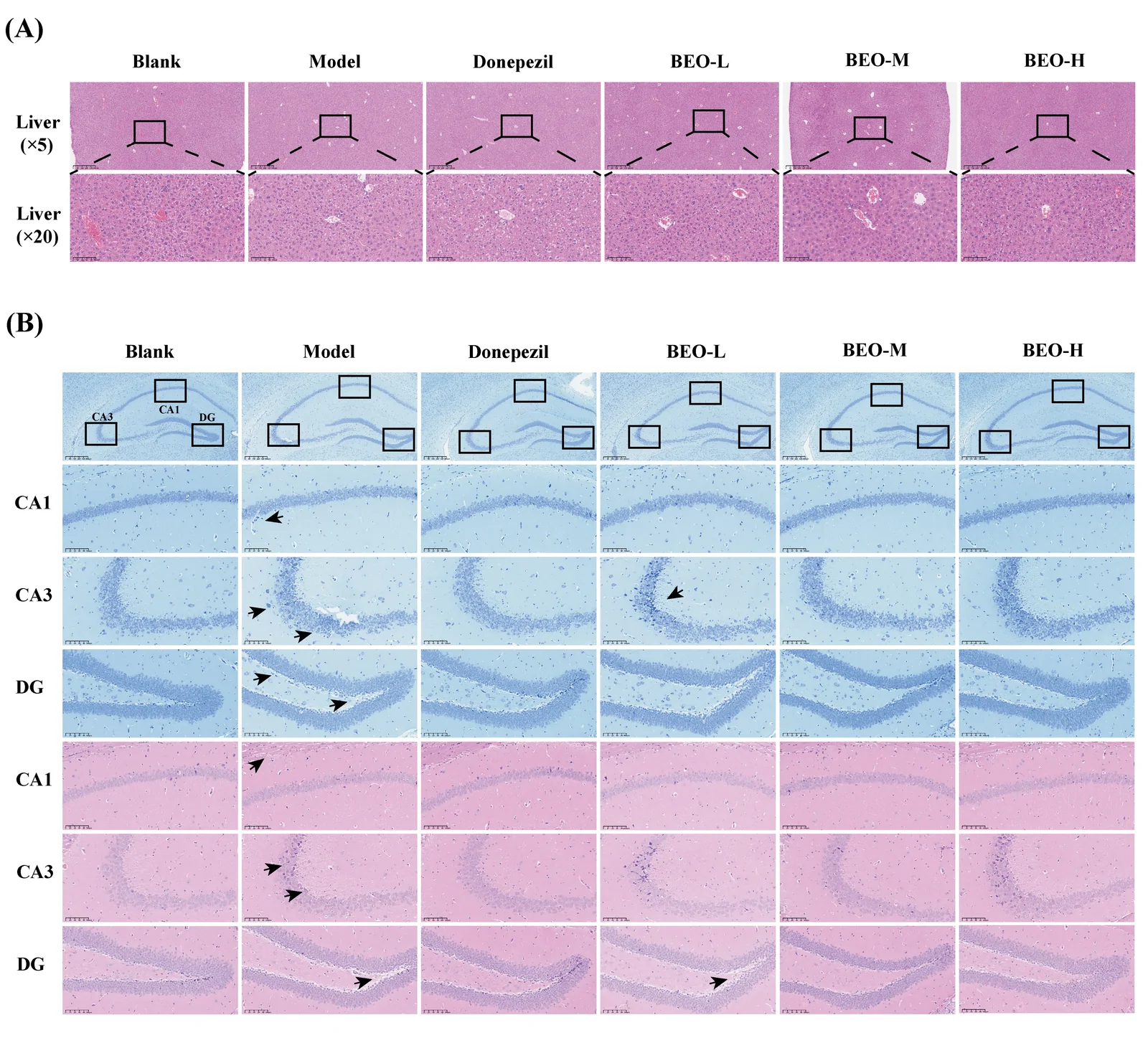
Liver biosafety assessment and hippocampal histopathological examination after BEO treatment. (**A**) Representative H&E staining of liver sections, shown at low (×5) and high (×20) magnification. (**B**) Representative hippocampal sections showing CA1, CA3, and DG regions by Nissl and H&E staining. Black arrows mark representative morphological abnormalities including neuronal shrinkage, cytoplasmic vacuolization, disorganized cellular lamination, and regions with reduced neuronal density. Blank group shows well-organized, intact hippocampal neuronal architecture. Qualitatively, donepezil and BEO administration appeared to preserve hippocampal cytoarchitecture and neuronal arrangement.

After performing H&E staining on the liver tissues of all groups, we observed that the morphology of the hepatocytes was quite normal, displaying polygonal shapes arranged in cords with clearly defined nuclei and uniform cytoplasm (Figure 3A). In none of the groups did we observe abnormal conditions such as inflammatory infiltration, necrosis, or steatosis. The findings indicated that the administration of BEO did not cause hepatotoxicity and confirmed that this treatment regimen was safely tolerated by the liver.

After 4 weeks of intranasal treatment with BEO, we performed H&E and Nissl staining on APP/PS1 mice to assess the integrity of hippocampal neurons (Figure 3B). In the Blank group, neurons were densely arranged, evenly distributed, and morphologically intact in the CA1, CA3, and DG regions. The boundaries between the cell nuclei and cytoplasm were clearly defined. In contrast, neurons in the Model group exhibited marked morphological damage, characterized by structural disorganization, cytoplasmic shrinking, vacuolization, and apparent reduction in neuronal density, which reflected severe degenerative changes in the hippocampus. Intranasal donepezil treatment effectively alleviated neuronal damage, restored orderly cell arrangement, improved neuronal density, and enhanced cell morphology. Hippocampal structures in mice administered low-dose, medium-dose, and high-dose BEO showed progressively improved outcomes. It is worth noting that medium- and high-dose BEO-treated groups exhibited qualitative relief of neuronal swelling, greater abundance of Nissl-stained cell bodies, and improved regularity and clarity of cell layer boundaries, suggestive of ameliorated neuronal morphological injury. The findings suggested that BEO can effectively reduce neuronal loss and protect neuronal function.

### 3.4. BEO Reduces Neuroinflammation in APP/PS1 Mice

**Figure 4:**
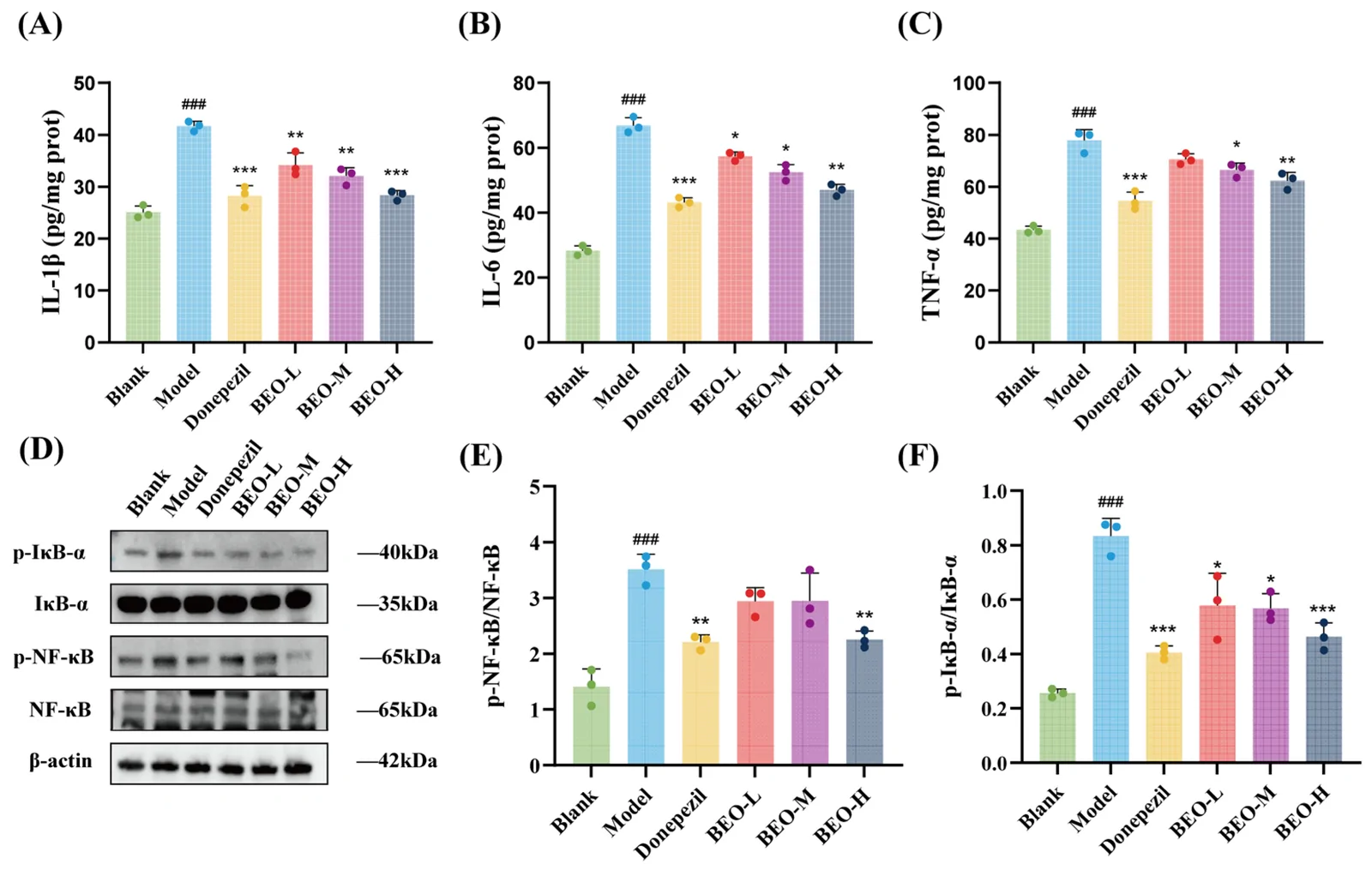
Intranasal BEO treatment suppressed neuroinflammatory cytokine production and was associated with reduced activation of the IκB-α/NF-κB signaling pathway in the AD model. (**A**–**C**) The Model group showed markedly increased hippocampal inflammatory cytokine levels compared with the Blank group, including IL-1β, IL-6, and TNF-α. Intranasal Donepezil and BEO treatment reduced these cytokines. (**D**) Representative Western blots of p-IκB-α/IκB-α, p-NF-κB/NF-κB, and β-actin. (**E**,**F**) Quantitative analysis showed that the Model group exhibited increased p-NF-κB/NF-κB and p-IκB-α/IκB-α ratios, indicating activation of the NF-κB inflammatory pathway. BEO-H significantly decreased p-NF-κB/NF-κB and p-IκB-α/IκB-α ratios, suggesting inhibition of IκB-α phosphorylation and downstream NF-κB activation. Data are presented as mean ± SD (*n* = 3). Statistical significance was determined by one-way analysis of variance followed by Tukey’s multiple-comparison test. ^###^ *p* < 0.001 vs. Blank; * *p* < 0.05, ** *p* < 0.01, and *** *p* < 0.001 vs. Model.

After undergoing intranasal BEO treatment for four weeks, we evaluated the levels of key pro-inflammatory cytokines and the activation of the IκB-α/NF-κB pathway in the hippocampus. Results from the ELISA indicated significantly higher levels of IL-1β, IL-6, and TNF-α in the Model group compared with the Blank group (*p* < 0.001) (Figure 4A–C), indicating a strong neuroinflammatory response. After intranasal treatment with donepezil and high-dose BEO, the levels of these cytokines decreased significantly (*p* < 0.001). Additionally, the cytokine levels in the BEO-M and BEO-L groups also decreased, but not as much. Greater effects were generally observed at higher BEO dose in the brains of APP/PS1 mice. Results of Western blot analysis demonstrated that, compared with the Blank group, the phosphorylation ratios of NF-κB (p-NF-κB/NF-κB) and IκB-α (p-IκB-α/IκB-α) were significantly elevated in the Model group (*p* < 0.001). This clearly indicated that the NF-κB signaling pathway has been activated (Figure 4D–F). Intranasal treatment with donepezil and BEO-H significantly reduced the p-NF-κB/NF-κB (*p* < 0.01) and p-IκB-α/IκB-α ratios (*p* < 0.001). Meanwhile, in the BEO-M and BEO-L groups, the inhibitory effects on these signaling markers were only partial. These findings suggested that BEO can weaken the expression of neuroinflammatory cytokines and regulate IκB-α/NF-κB signaling in the hippocampus. This highlights that BEO treatment was associated with reduced IκB-α/NF-κB pathway activation, alongside mitigation of AD-related inflammatory responses.

### 3.5. BEO Reduces Hippocampal Aβ Deposition in APP/PS1 Mice

**Figure 5:**
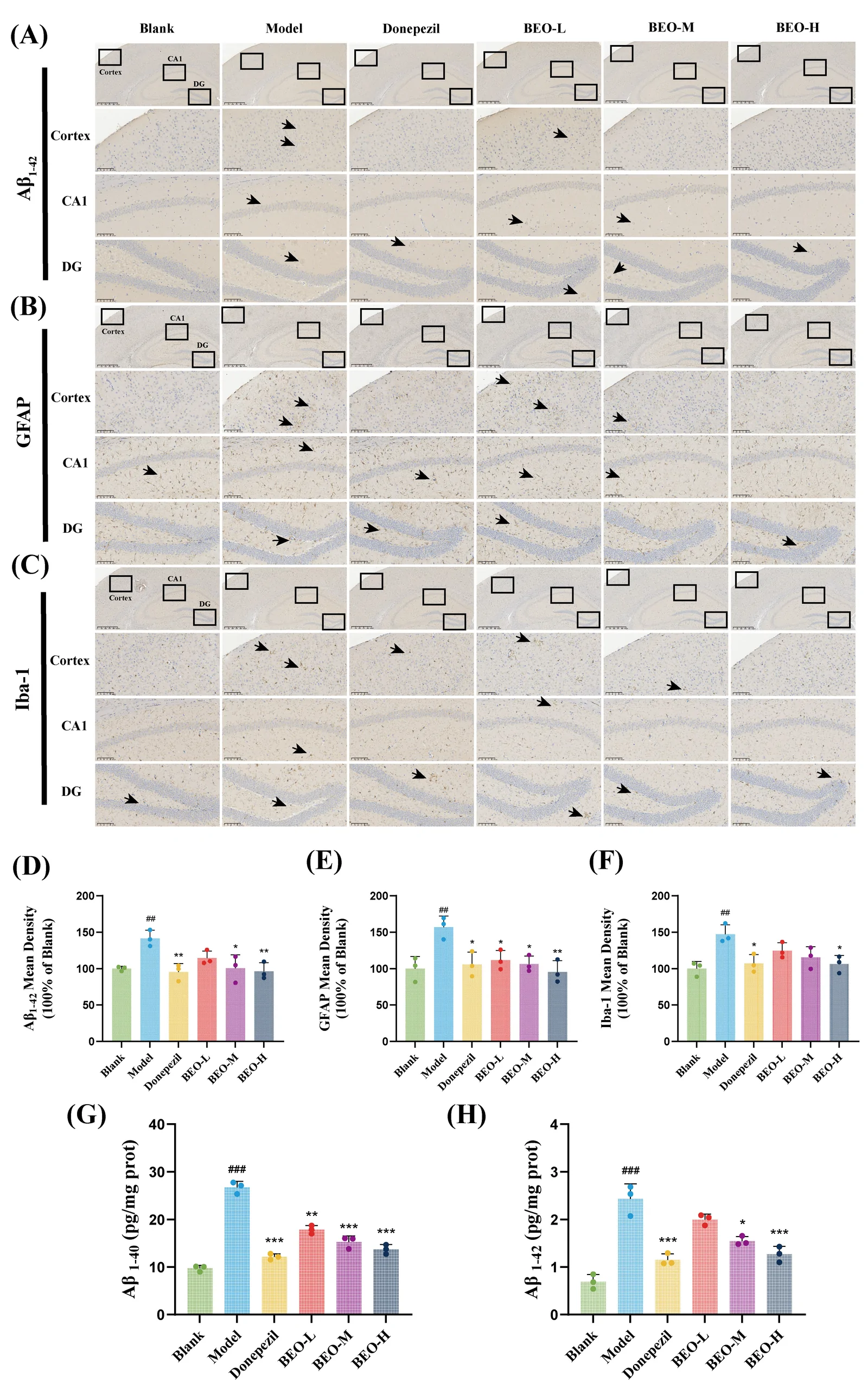
BEO reduces Aβ deposition and suppresses astrocytic and microglial activation within cortical and hippocampal regions. (**A**–**C**) Representative DAB immunohistochemical staining for Aβ_1–42_ (**A**), GFAP (**B**), and Iba-1 (**C**) in cortical and hippocampal CA1 and DG regions. Boxed areas in the low-magnification (×4) images are enlarged below (×20), and the arrows indicate representative positive staining. (**D**–**F**) Semi-quantitative analysis of immunohistochemical mean density, calculated as integrated optical density per tissue area and normalized to the Blank group (100%), for Aβ_1–42_ (**D**), GFAP (**E**), and Iba-1 (**F**). Compared with the Blank group, the APP/PS1 Model group showed significantly increased Aβ_1–42_ deposition and enhanced GFAP and Iba-1 immunoreactivity, indicating amyloid accumulation, astrogliosis, and microglial activation. Donepezil reduced all three IHC readouts. Intranasal BEO treatment lowered Aβ_1–42_ and glial-marker mean density, with BEO-M/BEO-H significantly reducing Aβ_1–42_, all BEO doses significantly reducing GFAP, and BEO-H significantly reducing Iba-1. (**G**,**H**) ELISA quantification of hippocampal Aβ_1–40_ (**G**) and Aβ_1–42_ (**H**) further confirmed elevated soluble amyloid levels in the Model group and their suppression by donepezil and BEO, particularly BEO-H. Multiple sections and microscopic fields originating from the same mouse were treated as technical replicates. Data are presented as mean ± SD (*n* = 3). Statistical significance was determined by one-way analysis of variance followed by Tukey’s multiple-comparison test. ^###^ *p* < 0.001, and ^##^ *p* < 0.01 vs. Blank; * *p* < 0.05, ** *p* < 0.01, and *** *p* < 0.001 vs. Model.

We evaluated the effects of BEO on Aβ accumulation in the hippocampus of APP/PS1 mice using ELISA and immunohistochemistry (Figure 5G,H). The results of ELISA showed that, compared with the Blank group, the levels of Aβ_1–40_ and Aβ_1–42_ were significantly elevated in the model group (*p* < 0.001), indicating the presence of extensive amyloid deposits. However, the situation improved significantly when donepezil or high-doses of BEO were administered to the mice. The concentrations of Aβ_1–40_ and Aβ_1–42_ in the hippocampal regions decreased significantly (*p* < 0.001). Both BEO-M and BEO-L groups exhibited a mild reduction. The results of the immunohistochemical staining of hippocampal and cortical sections were consistent with the ELISA data. Compared with the blank group, the accumulation of Aβ_1-42_ plaques was more pronounced in the model groups (Figure 5A,D).

### 3.6. BEO Attenuates Astrocyte and Microglial Activation in APP/PS1 Mice

Immunohistochemical analysis of the hippocampus and cortical tissues revealed that the Glial cells in APP/PS1 mice were significantly activated (Figure 5B,C,E,F). In the Model group, GFAP expression was markedly increased in CA1, CA3, DG, and cortical regions, indicating enhanced astrocyte activity. The immunoreactivity of Iba-1 also increased significantly in these areas, and microglia were more active than in the Blank group. Intranasal BEO treatment caused a decrease in the level of glial cell activation, and greater effects were generally observed at higher BEO doses. Low-doses of BEO decreased the expression of GFAP and Iba-1 slightly, while medium- to high-doses of BEO inhibited both astrocytes and microglia more effectively. Intranasal donepezil treatment also effectively reduced the expression of glial cell markers. These results suggested that BEO can suppress the activity of astrocytes and microglia in the hippocampus and cortical regions of APP/PS1 mice, which may help alleviate neuroinflammation associated with AD.

## 4. Discussion

The present study revealed that Xinjiang basil essential oil exerted broad neuroprotective effects in APP/PS1 mice. Multiple lines of corroborating evidence support these findings. UHPLC-MS/MS analysis identified a complex chemical profile, with a total of 787 compounds detected in BEO and 30 compounds detected in brain tissue following administration. In terms of effectiveness, intranasal administration of BEO improved spatial learning, memory retention, and exploratory activity. H&E staining of brain tissue confirmed that BEO reduced neuronal damage in the hippocampus. From a pathological perspective, BEO lowered the levels of Aβ_1–40_ and Aβ_1–42_ in the hippocampus, reduced the accumulation of Aβ_1–42_ plaques, and inhibited glial cell activation. In addition, BEO reduced inflammatory mediator generation and reduced activation of the IκB-α/NF-κB pathway. It seems that the effects of BEO were not limited to a single endpoint but rather exerted effects by modulating interrelated events involving amyloid deposition, neuroinflammation, and neuronal damage.

It is currently recognized that AD is caused by the combined action of multiple factors, including Aβ accumulation, tau hyperphosphorylation and tau-associated neurotoxicity, immune system dysregulation, synaptic dysfunction, metabolic stress, and progressive neurodegeneration [2]. In recent years, new therapies targeting amyloid have indeed been shown to modify this pathological process. However, clinical efficacy has remained limited in many patients, and safety concerns persist [20,21]. Consequently, approaches capable of simultaneously targeting multiple disease-related pathological processes are receiving increasing attention. Against this backdrop, the findings of our study hold significant importance. The BEO therapy not only reduced amyloid accumulation but also improved inflammation and neuronal function. This mechanism of action suggested that amyloid deposition and neuroinflammation may be mutually reinforcing processes rather than two independent pathological states.

A critical finding in this study was the detection of multiple putatively annotated features in brain homogenates after intranasal BEO administration. They were mainly composed of prenol lipids, with additional benzene/substituted derivatives, tannins, flavonoids, phenol ethers, organooxygen compounds, cinnamic acid derivatives, glycerophospholipids, and steroid-related compounds. This chemical diversity provides a possible basis for the multi-target biological activity observed in vivo. Previous studies have reported that *Ocimum* species and basil-derived preparations may improve cognitive decline and reduce oxidative stress or inflammatory injury [22,23]. Our previous network pharmacology study suggested that constituents from *Ocimum basilicum* L. may affect AD-related pathways, including AKT/GSK-3β signaling [24]. Our current results extend these observations by showing that BEO contains centrally accessible constituents and produces measurable behavioral, biochemical, and histological benefits in an AD mouse model. Rosmarinic acid stands out as a promising candidate supported by our analytical data and published evidence. Existing studies demonstrate its ability to inhibit neuroinflammatory signaling such as NF-κB activation and lower inflammatory mediators including TNF-α, IL-1β and IL-6. It can also reduce Aβ and phosphorylated tau accumulation and rescue cognitive deficits in 3×Tg-AD, alongside suppressed hippocampal inflammation and JNK signaling [25]. At the same time, the chemical complexity of BEO also means that the active compounds and their relative contributions and abundance remain to be defined. The constituents detected in brain tissue following intranasal administration may act additively or synergistically, so further characterization of BEO active components is required. Future pharmacokinetic studies, quantitative constituent analysis, receptor/enzyme assays, and loss-of-function or pathway-specific experiments will be needed.

The behavioral results support a functional benefit of BEO. APP/PS1 mice exhibited impairments in spatial learning and spatial reference memory in the MWM test and reduced exploratory behavior in the Open Field test. In the middle- and high-dose group, BEO decreased the escape latency, increased the number of platform crossings, and prolonged the time spent in the target quadrant as well as activity levels in the central region. The average swimming speed did not show any significant change, indicating that these effects were not solely due to improved athletic ability.

Histological observations were qualitatively consistent with the behavioral data. BEO treatment appeared to ameliorate neuronal morphological abnormalities including cytoplasmic shrinkage and vacuolation within hippocampal CA1, CA3, and DG regions. Nissl staining showed relatively preserved neuronal cell bodies and neuronal organization in the BEO-treated groups compared with the untreated APP/PS1 model group. Collectively, these data indicated that BEO-induced cognitive improvements were associated with the maintenance of hippocampal neuronal integrity.

BEO also reduced amyloid pathology. In APP/PS1 mice, levels of both Aβ_1–40_ and Aβ_1–42_ were significantly elevated. Immunostaining revealed dense plaque-like deposits throughout the hippocampus and cortex. Intranasal treatment with BEO reduced the level of soluble amyloid and significantly decreased the Aβ_1–42_ plaque burden. The therapeutic effect was generally more noticeable in the high-dose group. Aβ remains a primary pathological hallmark of AD and is also an important therapeutic target. Nevertheless, the amyloid burden depends on multiple processes, including APP processing, peptide aggregation, glial uptake, proteolytic degradation, vascular clearance, and interstitial fluid drainage [6,26]. Our study did not separate out individual mechanisms. Therefore, the reduction in Aβ levels following intranasal BEO treatment should be regarded as a combined pathological outcome. Future research will need to identify whether BEO alters the activity of β-secretase and γ-secretase, Aβ-degrading enzymes, microglial phagocytosis, or lymphatic clearance [27].

**Figure 6:**
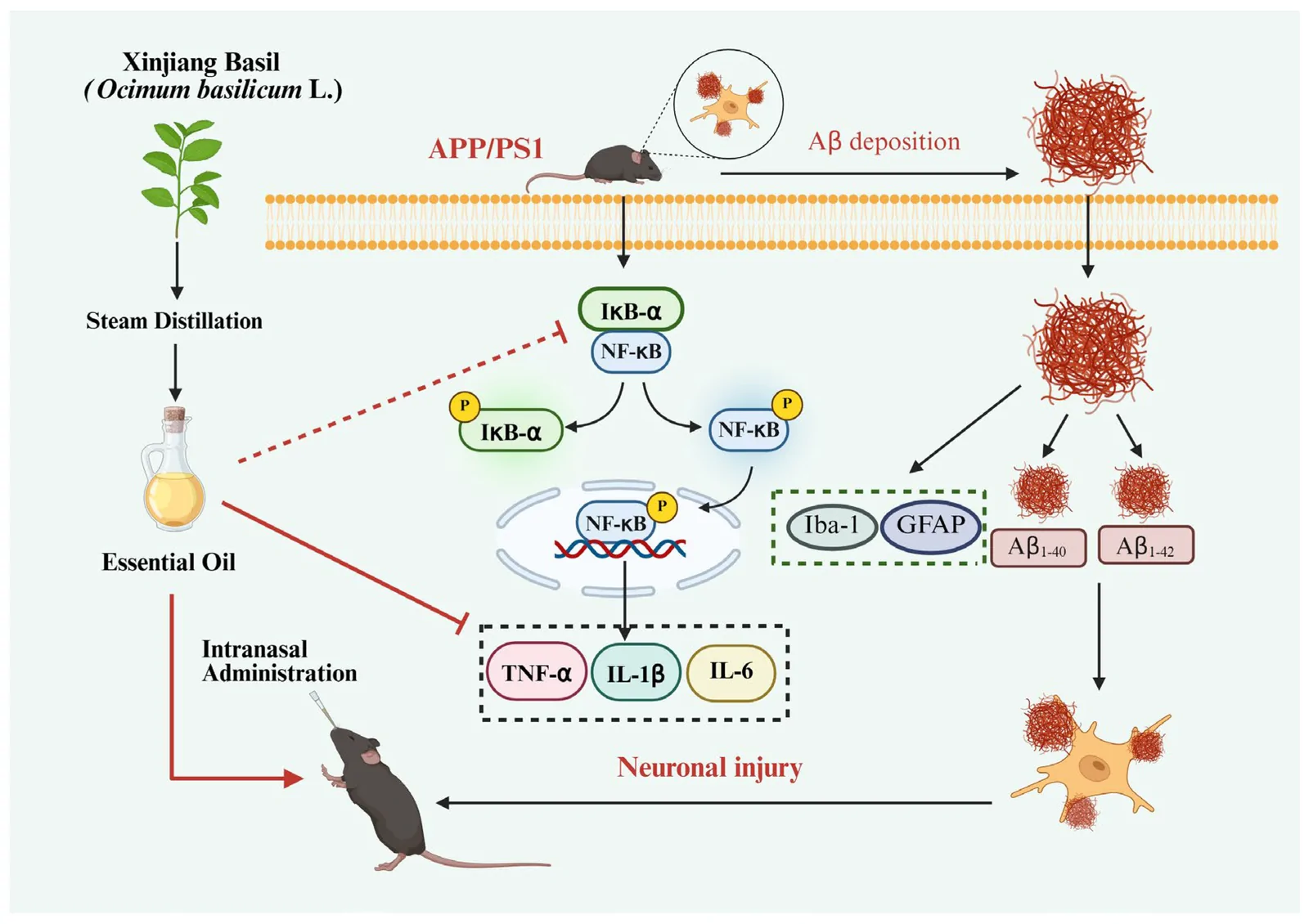
Proposed working model illustrating potential pathological inter-relationships after intranasal BEO treatment. Essential oil derived from Xinjiang Basil attenuates AD-like neuropathology-related readouts in APP/PS1 mice by suppressing Aβ deposition, neuroinflammation, and neuronal injury. Aβ accumulation promotes Aβ_1–40_/Aβ_1–42_ aggregation, activation of microglia and astrocytes, and induction of inflammatory mediators through the IκB-α/NF-κB signaling pathway. BEO inhibits IκB-α phosphorylation and NF-κB activation, thereby reducing downstream TNF-α, IL-1β, and IL-6 production, decreasing Iba-1 and GFAP associated glial activation, and ultimately alleviating neuronal injury and degenerative morphology. The exact route by which annotated features gain access to brain tissue (direct nose-to-brain versus systemic absorption across the blood–brain barrier) was not experimentally established in this work. Black arrows indicate pathological activation or progression; red blunt-ended lines indicate hypothesized inhibitory effects of BEO, causal validation via NF-κB pathway-perturbation assays is absent in the present study; yellow circles labeled “P” denote phosphorylation; red aggregates represent Aβ deposits.

Additionally, one of the key properties of BEO is its anti-inflammatory effect. In APP/PS1 mice, BEO reduced pro-inflammatory cytokine levels and lowered GFAP and Iba-1 immunoreactivity. These changes indicated elevated inflammation in the mice and the activation of astrocytes and microglia, which play important roles in the neuroinflammation associated with AD. However, it should be noted that the activation of glial cells is not always detrimental. Astrocytes and microglia can promote Aβ clearance and tissue repair. Nevertheless, prolonged activation may intensify cytokine production, synaptic dysfunction, and neuronal loss [9,28]. In this study, BEO reduced the mean density of GFAP and Iba-1 and also decreased the levels of inflammatory cytokines, indicating that it can suppress excessive glial activation. This conclusion is further supported by the decrease in the ratios of p-NF-κB/NF-κB and p-IκB-α/IκB-α. The NF-κB signaling pathway is a critical regulatory factor for the expression of cytokines and is closely related to neurotoxicity in models of neurodegenerative diseases [29]. However, our observations only revealed a reduced correlation with the activation level of the IκB-α/NF-κB pathway in animals administered BEO. Definitive causal evidence demonstrating that modulation of this axis mediates BEO-driven protective phenotypes awaits targeted pathway-perturbation experiments. The proposed working model was illustrated in Figure 6.

Differential effects across BEO dose groups further reinforces the biological plausibility of the findings. BEO-H group showed the most significant and stable effects in improving behavioral performance, reducing Aβ burden, lowering inflammatory marker levels, alleviating glial cell activation, and reducing the activation of IκB-α/NF-κB signaling pathway. BEO-M also improved several indicators, while the BEO-L group had slightly weaker effects, but still showed detectable benefits. Donepezil was selected as the positive-control drug because it is an established pharmacological treatment for symptomatic cognitive impairment in AD and acts primarily as an AChE inhibitor, thereby increasing the availability of acetylcholine in the synaptic cleft [30]. The purpose of including donepezil was not to use it as a mechanistic control for the NF-κB pathway, but rather as a clinically established cognitive-enhancing comparator to confirm the responsiveness of the experimental model and behavioral paradigm. As a positive control, Donepezil improved cognitive function and reduced several pathological markers, which in turn proves the reliability of this model and experimental protocol.

Basil has a long history of use as an edible culinary herb and flavoring agent, and available toxicological evidence generally supports the safety of basil consumed as a food or used in conventional preparations. Experimental toxicological evidence generally supports the safety of basil-derived preparations [31]. A toxicological study on basil leaves at three different growth stages reported that basil leaf extracts did not show potential toxicity [32]. However, this favorable dietary safety profile should not be directly extrapolated to concentrated essential oils, particularly when administered intranasally. Essential oils contain highly concentrated volatile constituents and therefore require dedicated toxicological evaluation. In the present study, no obvious hepatotoxic morphological changes, including inflammatory infiltration, necrosis, or steatosis, were observed after 4 weeks of BEO administration. These findings provide preliminary evidence that the present treatment regimen was tolerated by the liver. Nevertheless, the current study was not designed to establish the long-term safety of intranasal BEO. Future studies should systematically evaluate chronic toxicity, nasal mucosal tolerance, hematological and serum biochemical parameters, pharmacokinetics, and potential target-organ toxicity following prolonged administration.

The following limitations must be taken into account. First, APP/PS1 mice primarily mimic the disease progression driven by β-amyloid but fail to fully replicate the tau pathology, neural network dysfunction, and clinical heterogeneity observed in human AD [33,34,35]. Second, with relatively short intervention periods, the long-term protective effects remain unclear. Third, insufficient sample sizes for biochemical and histological assays might compromise the statistical validity of the results. Fourth, although immunohistochemistry provided valuable semi-quantitative information, it failed to determine the specific phenotype or cellular functional state. Co-localization staining, flow cytometry, spatial transcriptomics, and single-cell sequencing are among the techniques that help us better understand the effects of BEO on microglial and astrocyte subtypes [36]. While 30 constituents detected in brain tissue following intranasal administration were identified, the active ingredients, targets, and potential synergistic mechanisms of these compounds still need to be validated. These issues are critical to resolve prior to advancing BEO into the phase of translational research and product development. In addition, Tween 80 is a polyoxyethylene derivative of a nonionic surfactant. Although several in vivo studies have shown that intranasal administration of Tween 80 not only exhibits good biocompatibility but also acts as an effective absorption enhancer and targeting agent [37,38,39], this study used Tween 80 without establishing a solvent control group to verify its impact on the main experiment.

Research in the future will focus on three areas. The first is to conduct pharmacokinetic and pharmacodynamic analyses to determine which constituents detected in brain tissue following intranasal administration, their residence time in the brain, and the concentrations required to produce an effective effect. Future pharmacokinetic studies, GC-MS/MS quantitative constituent analysis, receptor/enzyme assays, and loss-of-function or pathway-specific experiments will be needed. Additionally, we should perform mechanistic experiments to validate the effects of BEO on Aβ generation and clearance, glial phagocytosis, synaptic protection, mitochondrial function, and NF-κB-dependent transcription [40]. Future work should include NF-κB activator co-treatment rescue experiments and cell-based pathway manipulation assays to test whether NF-κB-axis modulation is causally required for the protective effects of BEO. Without such perturbation evidence, our current interpretation remains limited to correlative associations in vivo. Finally, we need to validate the efficacy of BEO in other AD models, such as those involving tau pathology and elderly mice. This will help determine if BEO’s effects are limited to amyloid-related processes [41,42,43]. These studies are crucial for elucidating the role of BEO as a preventive nutritional foods, adjunctive therapy, or source of lead compounds for drug development in AD.

## 5. Conclusions

To summarize, this study indicated that Xinjiang basil essential oil contains multiple constituents detected in brain tissue following intranasal administration and exhibiting neuroprotective effects in the APP/PS1 mice. Intranasal administration of BEO enhanced cognitive function, preserved hippocampal neuronal structure, reduced the accumulation of Aβ_1–40_ and Aβ_1–42_, suppressed microglial and astrocyte activation, decreased inflammatory cytokine generation, and BEO treatment was associated with reduced activation of the IκB-α/NF-κB signaling pathway. Together, these findings indicated that the BEO attenuated the AD-like pathology through regulating amyloid burden and neuroinflammation. Further research remains necessary to identify active components, clarify direct molecular targets, and verify long-term safety. Nonetheless, BEO demonstrated promising potential as a natural, multi-component intervention for treating AD-related neurodegenerative disorders.

## Figures and Tables

**Table 1:** Source gas parameters for the UHPLC–TripleTOF 6600.

Parameters	Set-Point
Ion Spray Voltage	+5500/−4500 V
Temperature	500 °C
Ion Source Gas1	60 psi
Ion Source Gas2	60 psi
Curtain Gas	45 psi
Declustering Potential	60 V
Tof Ms Scan CE	10 V
Product Ion Scan CE	40 V
Collision Energy Spread	20 V

**Table 2:** 30 putatively annotated compounds were detected in brain tissue after intranasal administration of BEO.

No.	RT	Ion Mode	Identification	*m*/*z*	Formula	Class
1	5.772	[M ^+^ H]^+^	(-)-Carvone	151.11078	C_10_H_14_O	Prenol lipids
2	3.649	[M ^+^ H]^+^	(R)-(+)-Pulegone	153.12793	C_10_H_16_O	Prenol lipids
3	3.774	[M ^−^ H]^−^	1,2,3,6-Tetragalloylglucose	787.09497	C_34_H_28_O_22_	Tannins (putative annotation) ^1^
4	3.548	[M ^−^ H]^−^	1,2,3-Tri-O-galloyl-beta-D-glucose	635.0799	C_27_H_24_O_18_	Tannins (putative annotation) ^1^
5	13.343	[M ^−^ H]^−^	1-18:1-lysoPE	478.29105	C_23_H_46_NO_7_P	Glycerophospholipids (putative annotation) ^1^
6	3.593	[M ^+^ H]^+^	2,4-Hexadienal	97.06488	C_6_H_8_O	–
7	4.773	[M ^−^ H]^−^	2-Methyltryptoline	185.11566	C_12_H_14_N_2_	–
8	3.602	[M ^+^ H]^+^	4-Isopropyl-3-methylphenol	151.11238	C_10_H_14_O	Benzene and substituted derivatives
9	4.082	[M ^−^ H]^−^	Acetosyringone	195.06506	C_10_H_12_O_4_	Organooxygen compounds
10	4.403	[M ^+^ H]^+^	Angelin	367.21677	C_24_H_30_O_3_	Prenol lipids
11	14.772	[M ^−^ H]^−^	Ardisenone	495.26929	C_30_H_40_O_6_	Organooxygen compounds
12	3.597	[M ^+^ H]^+^	Asarone	209.1136	C_12_H_16_O_3_	Phenol ethers
13	5.892	[M ^−^ H]^−^	Calyxanthone	285.0441	C_15_H_10_O_6_	–
14	3.868	[M ^+^ H]^+^	Cinchonine	295.18579	C_19_H_22_N_2_O	Cinchona alkaloids (putative annotation) ^1^
15	15.062	[M ^−^ H]^−^	Epijuvabiol	267.19485	C_16_H_28_O_3_	Prenol lipids
16	4.662	[M ^+^ H]^+^	Evodol	485.17075	C_26_H_28_O_9_	Steroids and steroid derivatives
17	4.887	[M ^+^ H]^+^	Geranic acid	169.12097	C_10_H_16_O_2_	Prenol lipids
18	4.045	[M ^+^ H]^+^	Kaempferol 3-(6″-malonylglucoside)	535.10938	C_24_H_22_O_14_	Flavonoids (putative annotation) ^1^
19	3.604	[M ^−^ H]^−^	Luteolin 4′-glucoside	447.09012	C_21_H_20_O_11_	Flavonoids (putative annotation) ^1^
20	13.045	[M ^−^ H]^−^	Lythranidine	424.24258	C_26_H_35_NO_4_	Phenol ethers
21	3.868	[M ^+^ H]^+^	Norbergenin	315.06766	C_13_H_14_O_9_	Benzene and substituted derivatives
22	14.556	[M ^+^ Na]^+^	Oleanolic acid	479.35553	C_30_H_48_O_3_	Prenol lipids
23	5.89	[M ^+^ H]^+^	Pedicinin	301.06824	C_16_H_12_O_6_	Prenol lipids
24	4.25	[M ^−^ H]^−^	Rosmarinic acid	359.07501	C_18_H_16_O_8_	Cinnamic acids and derivatives
25	3.636	[M ^+^ H]^+^	Thalrugosaminine	653.33496	C_39_H_44_N_2_O_7_	Tannins (putative annotation) ^1^
26	4.081	[M ^+^ H]^+^	Viscumamide	566.42285	C_30_H_55_N_5_O_5_	—
27	10.832	[M ^+^ H]^+^	Z-gamma-Atlantone	219.17424	C_15_H_22_O	Prenol lipids
28	3.6	[M ^+^ H]^+^	alpha,4-Dimethylstyrene	133.10046	C_10_H_12_	Benzene and substituted derivatives
29	3.585	[M ^−^ H]^−^	alpha-Viniferin	677.18433	C_42_H_30_O_9_	—
30	9.903	[M ^−^ H]^−^|[M ^+^ H]^+^	Chrysanthemic acid	167.10889|169.12256	C_10_H_16_O_2_	Prenol lipids

^1^ All compounds listed represent putative annotations derived from LC-MS/MS spectral matching in brain homogenate samples after intranasal BEO administration. None of these annotations were confirmed via authentic reference standards. Some annotated molecules exhibit high molecular weight or high polarity, which are chemically atypical for steam-distilled volatile essential-oil constituents.

## Data Availability

The data are contained within the article.

## Abbreviations

The following abbreviations are used in this manuscript:

Aβ	Amyloid-β
AChE	acetylcholinesterase
AD	Alzheimer’s disease
BEO	Basil essential oil
DG	Dentate gyrus
H&E	Hematoxylin–Eosin
IDA	Information-dependent acquisition
IHC	Immunohistochemistry
MWM	Morris Water Maze
SD	Standard deviation
SPF	Specific Pathogen-Free
TOF MS	Time-of-flight Mass Spectrometry
